# Resection of liposarcoma of the greater omentum: A case report and literature review

**DOI:** 10.1016/j.ijscr.2019.06.067

**Published:** 2019-07-08

**Authors:** Shintaro Hashimoto, Junichi Arai, Masato Nishimuta, Hirofumi Matsumoto, Hidetoshi Fukuoka, Masashi Muraoka, Masahiro Nakashima, Hiroyuki Yamaguchi

**Affiliations:** a Department of Surgery, Japan Community Health care Organization (JCHO), Isahaya General Hospital, Japan; b Department of Pathology, Japan Community Health care Organization (JCHO), Isahaya General Hospital, Japan

**Keywords:** Omental liposarcoma, Omental tumour, Liposarcoma

## Abstract

**Introduction:**

Liposarcoma usually occurs in the retroperitoneum and limbs. Liposarcoma of the greater omentum is rare, and most information of such liposarcomas has come from case reports.

**Presentation of case:**

A 60-year-old woman was found to have an 8-cm intra-abdominal mass (suspected lipoma) by computed tomography. At the age of 63 years, she underwent a medical examination and a mass was palpated in the abdomen. Contrast-enhanced computed tomography and magnetic resonance imaging confirmed the presence of a huge intra-abdominal tumor with the omental artery passing through the mass. The tumor was simply resected. Histopathologically, the tumor was diagnosed as a well-differentiated liposarcoma, and the resection margin was microscopically negative. The patient had developed no recurrence or complications 9 months postoperatively.

**Discussion:**

Liposarcoma of the greater omentum is rare, and differentiation of liposarcoma from other tumors is challenging. Adjuvant therapy has not been established as an effective treatment, and radical (R0) resection of the tumor is recommended. Our case of liposarcoma of the greater omentum was surgically managed with good outcomes.

**Conclusion:**

The diagnosis of liposarcoma with a lipomatous tumor is challenging, and resection should be considered for huge intra-abdominal lipomatous tumors.

## Highlights

• Intraabdominal liposarcoma including omental liposarcoma is rare.
• Liposarcoma can be difficult to distinguish from other lipomotous tumor.
• We surgically managed a case of liposarcoma of omentum ovserved as lipoma 3 years ago.

## 1 Introduction

Liposarcoma is one of the most common soft tissue sarcomas. It accounts for approximately 10% of all soft tissue sarcomas, and its peak incidence occurs around the fifth to sixth decades of life [1]. Intra-abdominal liposarcoma, including omental sarcoma, is rare [2]. We herein report a surgical case of liposarcoma of the greater omentum along with a review of the literature. It is reported in line with the PROCESS criteria [3].

## 2 Presentation of case

**Fig. 1:**
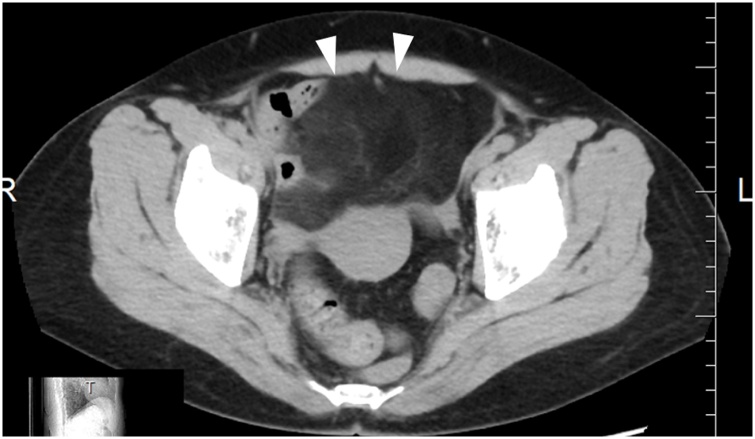
Abdominal CT showed an 8 × 8 × 4 cm mass (arrowhead) on the cranial side of the bladder in the abdomen.

A 60-year-old woman underwent screening blood tests, which revealed a high serum amylase level. Computed tomography (CT) showed an 8- × 8- × 4-cm abdominal mass on the cranial side of the bladder, an intra-abdominal lipoma was suspected and observed (Fig. 1).

**Fig. 2:**
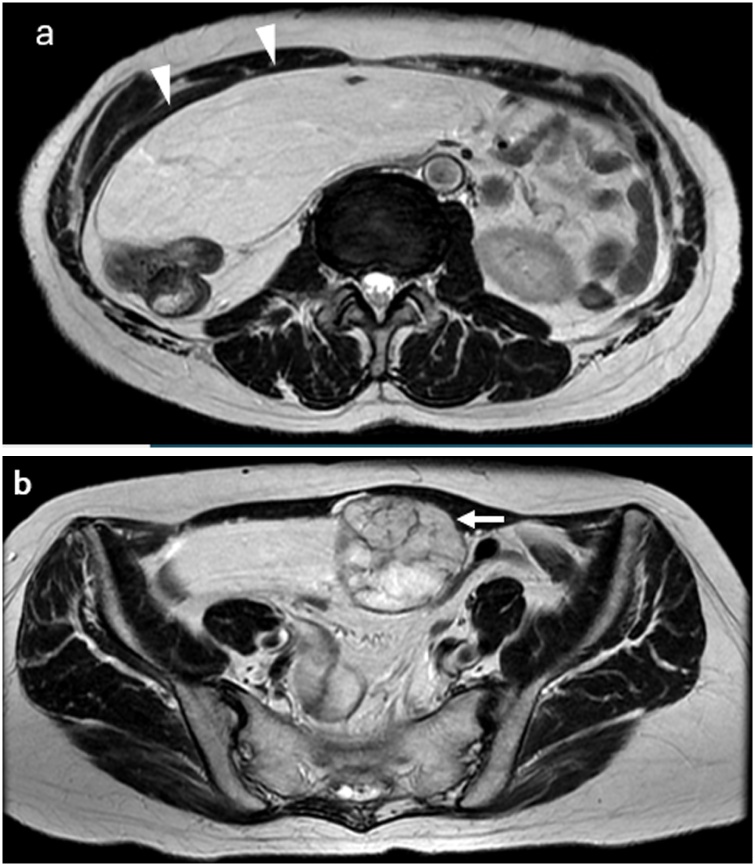
(a) T2-weighted MRI showed a large, well-defined, low-signal mass measuring 20 × 17 × 7 cm (arrowhead) in the abdomen. (b) The mass was adjacent to another abnormal region measuring 6 × 6 × 5 cm with septae and a capsule (arrow).

Three years later, she underwent a medical examination, and an elastic hard mass with slight tenderness was palpated in the abdomen. She had no abdominal pain, nausea, constipation, or other symptoms. Contrast-enhanced CT and magnetic resonance imaging (MRI) showed a large, well-defined abdominal mass with low attenuation and fat density measuring 20 × 17 × 7 cm. The mass was adjacent to another abnormal region measuring 6 × 6 × 5 cm with septae and a capsule. Contrast-enhanced CT also revealed omental artery involvement in the mass, and an omental tumor was suspected. MRI showed no evidence of invasion to other organs, including the digestive tract, bladder, or great vessels. A liposarcoma (smaller region) with a lipoma (larger mass) was suspected, and no metastatic lesions were observed (Fig. 2a, b). After the conference of the surgeons and physicians, confirming the possibility of the tumor resection being with other structures in case of invasion, the patient consented the plan for the surgery and surgical resection was performed.

**Fig. 3:**
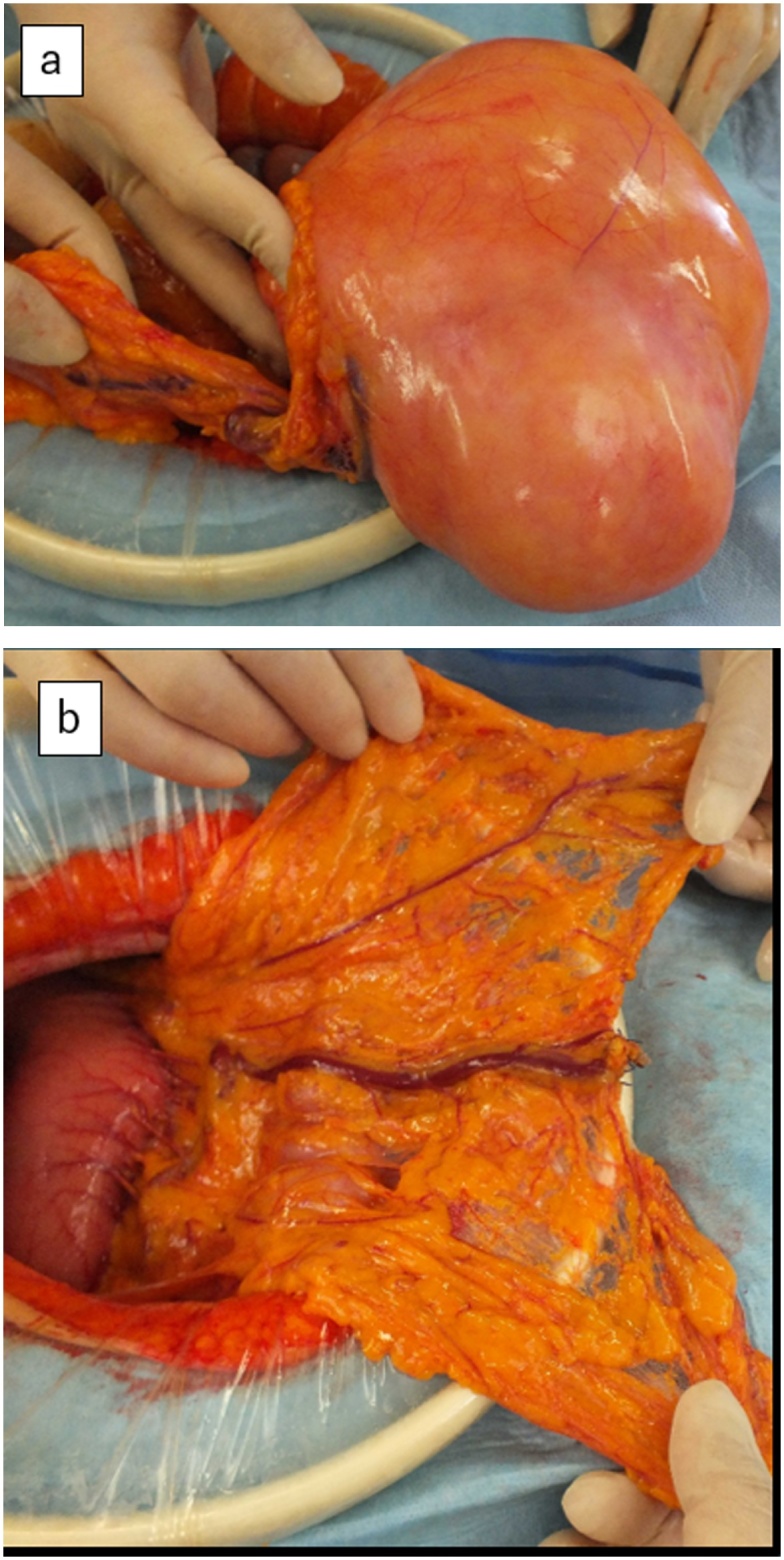
(a) A huge yellowish, soft mass with a dark reddish-gray region adjacent to the mass was located under the abdominal wall. (b) The feeder vessel originated from the omental artery.

Intraoperatively, 20 cm midline incision of the abdominal wall was made. A huge, yellowish soft mass with a dark reddish-gray region adjacent to the mass was found under the abdominal wall without invasion, including colon, intestine, mesentery, abdominal wall, bladder, uterus, and retroperitoneum. After ligation of the feeder vessel originating from the omental artery, existing cranial side of the mass, the mass was resected en bloc (Fig. 3a, b). No evidence of intra-abdominal metastasis was found. The surface of the mass was carefully treated and not raptured.

**Fig. 4:**
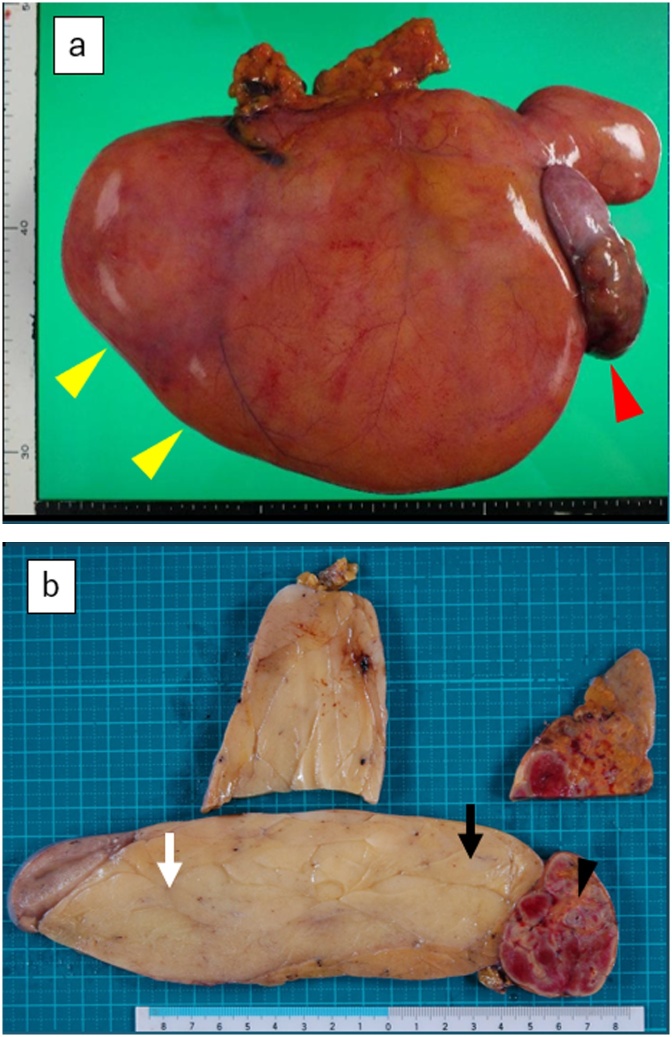
(a) The resected specimen, including the yellowish mass (yellow arrowhead) and reddish-gray region (red arrowhead), weighed 3750 g and measured 27 × 20 × 10 cm. (b) The cut surface of the tumor was shown. The diagnosis was a well-differentiated liposarcoma in the areas of black arrow and black arrowhead, and a lipoma in the area of white arrow.

**Fig. 5:**
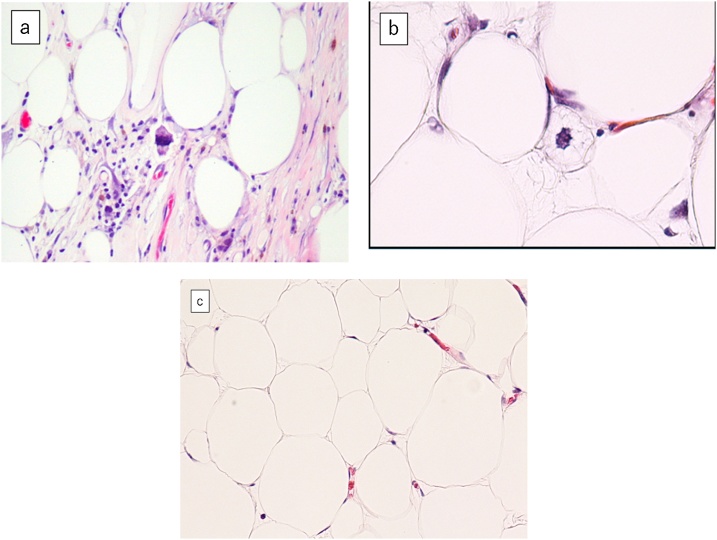
(a) Neoplastic spindle cells with atypical nuclei containing condensed chromosomes were present in the septae in the reddish-gray region (black arrowhead in Fig. 4b). (b) Malignant cells were also seen in the area near the reddish-gray region in the yellowish mass (black arrow in Fig. 4b). (c) The area far from the reddish-gray region in the yellowish mass (white arrow in Fig. 4b) was composed of mature adipocytes with uniform nuclei resembling normal fat tissue.

The resected specimen, which comprised a yellowish mass and reddish-gray region, weighed 3750 g and measured 27 × 20 × 10 cm (Fig. 4a, b). Histopathological examination showed that within the reddish-gray region (black arrowhead in Fig. 4b), neoplastic spindle cells with atypical nuclei containing condensed chromosomes were present in the septae (Fig. 5a). Lipoblasts and inflammatory cells were present. Necrosis of fat was also observed. Near the reddish-gray region in the yellowish mass (black arrow in Fig. 4b), malignant cells were also seen (Fig. 5b). Far from the reddish-gray region in the yellowish mass (white arrow in Fig. 4b), mature adipocytes with uniform nuclei resembling normal fat tissue were observed (Fig. 5c). Immunohistochemical analysis revealed the MDM2+/CDK4+ immunophenotype (black arrowhead and black arrow in Fig. 4b) and the MDM2−/CDK4− immunophenotype (white arrow in Fig. 4b).

The diagnosis was a well-differentiated liposarcoma (black arrow and black arrowhead in Fig. 4b) and a lipoma (white arrow in Fig. 4b) based on the histopathological features. The resected margin was microscopically negative (R0).

The patient had an uneventful postoperative period. No recurrence or significant problems had occurred at 9 months postoperatively.

## 3 Discussion

Liposarcomas are usually located in the gluteal region, thighs, popliteal fossa, shins, and retroperitoneum [4]. Intra-abdominal liposarcoma is uncommon [2]. In addition to the greater omentum, liposarcomas have been reported in the small bowel [5], small bowel mesentery [6], colon [7], and mesorectum [8]. Omental metastatic and recurrent tumors are common, but primary tumors are relatively rare [9]. Primary tumors of the greater omentum have various histological differential diagnoses including liposarcoma, lipoma, mesothelioma, hemangiopericytoma, stromal tumor, leiomyoma, neurofibroma, fibroma, fibrosarcoma, and leiomyosarcoma [10].

The prognosis of a liposarcoma in the trunk, including the intra-abdominal region, retroperitoneum, and thoracic cavity, is worse than that of a liposarcoma in the extremities. In one study, the median 5-year disease-free survival rates for liposarcomas in the trunk vs. extremities were 41.9% vs. 66.7% (P < 0.001), and the 5-year overall survival rates were 64.5% vs. 84.5% (P < 0.001), respectively [11]. The prognosis of these tumors also depends on the histological subtype. The 5-year disease-free survival rate is worse for dedifferentiated, round cell, and pleomorphic liposarcomas (high-grade group) than for well-differentiated and myxoid types (low-grade group) (16.9% vs. 65.7%, P < 0.001); the 5-year overall survival rate is also worse (47.8% vs. 83.5%, P < 0.001) [11,12].

Primary omental liposarcoma is rare, and De et al. [13] reviewed nine cases from 1936 to 2003. Since that review, 10 cases were reported in the English-language literature from 2003 to 2018, including our case. These 19 cases were reviewed in the present study (Table 1). The average age of the patients was 51.1 years (range, 11–83 years). Although our patient was asymptomatic, the patients in previous reports exhibited various symptoms including abdominal pain, swelling, fever [13], constipation [14], and abdominal distention [15]. Cases of liposarcoma of the greater omentum presenting as inguinal hernia and torsion have also been reported [16]. Our patient developed ischemic change in the reddish-gray, elastic hard region of the mass. We considered that this ischemic mass may have developed by torsion; nevertheless, the patient had no episodes of acute abdominal pain. Complete tumor resection is recommended for greater omental liposarcoma [16]. Our review showed that the tumors in 15 patients (78.9%) were resectable and that wide tumor resection was needed for 4 patients (26.7%). In terms of histologic subtypes, three tumors (15.8%) were well-differentiated, five (26.3%) were myxoid, three (15.8%) were pleomorphic, four (21.1%) were round cell, one (5.3%) was dedifferentiated, and three (15.8%) were not classified. Although our review of these 19 cases included a long-term survivor (13 years) with myxoid liposarcoma, 6 patients died during the follow-up period. A recent study suggested that postoperative radiation therapy may improve outcomes in patients with retroperitoneal liposarcoma, especially for subtypes other than well-differentiated tumors [17]. Because of the risk of radiation enteritis and the rarity of the disease, adjuvant radiation for omental liposarcoma remains controversial. Although adjuvant chemotherapy also remains controversial, chemotherapy seems promising in the treatment of liposarcoma [[18], [19], [20]].

**Table 1:** The characteristics of the reported cases of a liposarcoma of the greater omentum.

Reference	No.	Author/year	Age/Sex	Main clinical presentation	Preoperative imaging	Operation	Torsion	Weight (g)	Size(cm)	Histological subtype	Adjuvant therapy	Follow- up	Outcome
[27]	1	Manne et al/1936	40/M	Abdominal swelling	Unknown	Repeated paracentesis	Unknown	Unknown	Unknown	Not classified	Unknown	9 month	Dead
[28]	2	Robb/1960	34/M	Epigastric pain	Unknown	Resection (details unknown)	Unknown	Unknown	Unknown	Not classified	Unknown	6 month	No recurrence
[29]	3	Stout et al/1963	60/F	Abdominal pain and swelling	Unknown	Laparotomy and biopsy	Unknown	Unknown	Unknown	Myxoid	Unknown	2 days	Dead
[30]	4	McAvoy et al/1978	65/M	Abdominal distension	Unknown	Resection (details unknown)	Unknown	Unknown	Unknown	Not classified	Yes	Not mentioned	Not mentioned
[31]	5	Kadow et al/1989	36/M	Abdominal distension and dyspepsia	US	Simple tumor resection	No	Unknown	25 × 19 × 15	Pleomorphic	Radiation + CPA + VCR + ADM + dacarbazine	3 months	Local recurrance
[31]	6	Kadow et al/1989	71/M	Retrosternal discomfort, lethargy, weight loss, night sweats	Unknown	Simple tumor resection	No	Unknown	28 × 18 × 12	Pleomorphic	No	Not mentioned	Not mentioned
[3]	7	Okajima et al/1993	54/F	Abdominal swelling, leg edema	US, CT, angipography	Simple tumor resection	Unknown	2,300	27 × 17 × 11	Round cell	No	10 months	No recurrence
[15]	8	Tsutsumi et al/1999	83/M	Abdominal pain and distention	US, CT, angipography	Simple tumor resection	Yes	640	18 × 10 × 7	Round cell	No	2 years	No recurrence
[12]	9	De et al/2003	45/M	Abdominal pain and distention, fever	US, CT	Simple tumor resection	No	950	15 × 10 × 2, with smallnodules	Round cell	No	9 months	Dead
[32]	10	Alameda et al/2003	25/F	Abdominal distention	CT	Wide tumor resection with epiploic appendices	No	2,100	24 × 24 × 4	Round cell	No	1 year	Survival with no information of recurrance
[33]	11	Milic et al/2004	50/F	Abdominal distension and costipation	Not mentioned	Simple tumor resection	No	1,900	22 × 12 × 7	Myxoid	No	13 years	Dead (Peritoneal dissemination)
[2]	12	Milic et al/2005	52/M	Left abdominal and groin pain	Not mentioned	Simple tumor resection	No	1,400	17 × 11 × 7	Myxoid	No	3.5 years	No recurrence
[34]	13	Imai et al/2006	55/F	Weight loss and abdominal distension	US, CT	Volume reduction surgery	No	5,900	Over 15 cm (US)	Myxoid	No	1 month	Dead (Progress of primary tumor)
[20]	14	Meloni et al/2009	34/M	Abdominal distension	US, CT, MRI	Resection (details unknown)	No	Unknown	25 × 13 (CT)	Well- differentiated	No	5 years	No recurrence
[14]	15	Soufi et al/2012	65/F	Abdominal pain and distension, constipation	CT	Wide tumor resection with omentectomy, appendectomy	No	Unknown	30 × 27 × 19 (CT)	Dedifferentiated	Doxorubicin	8 months	No recurrence
[35]	16	Tomita et al/2012	63/M	Abdominal discomfort and fever	CT	None	No	Unknown	Not mentioned	Pleomorphic	No	2.5 months	Dead (Lung congestion and pneumonia)
[13]	17	Hightower et al/2014	11/M	Abdominal pain	US, CT	Wide tumor resection with appendectomy	No	4,500	21 × 8×8 (CT)	Myxoid	No	6 months	Lung metastases
[17]	18	Rajshekher/2015	65/F	Abdominal pain and distension, decreased appetite	US, CT	Wide tumor resection with omentectomy, wedge resection of stomach	No	7,500	23 × 20 × 12	Well- differentiated	Doxorubicin	3 years	No recurrence
	19	Our case/2018	63/F	Without symptoms (health check)	CT, MRI	Simple tumor resection	Yes	3,750	27 × 20 × 10	Well- differentiated	No	9 months	No recurrence

US: Ultra sonography.

CT: Computed tomography.

MRI: Magnetic Resonance Imaging.

CPA: Cyclophosphamide.

VCR: Vincristine.

ADM: Adriamycin.

Liposarcoma often has different histological components including both benign and malignant areas [21,22]. The tumor in the present case had two sections: a reddish-gray area with ischemic change and a huge, soft fatty yellowish mass. Liposarcoma was diagnosed in the reddish-gray area, and the near side of the fatty yellowish mass was formed by lipoblasts. Mature fat cells were observed in the far side of the yellowish mass, and this region was diagnosed as lipoma. A recent study suggested biologic potency of transformation of benign lipoma into well-differentiated liposarcoma [23,24]. Nevertheless, the pathogenetic concept of liposarcoma arising from benign lipoma is generally not accepted [24,25].

The CT and MRI appearances of a well-differentiated liposarcoma are similar to those of normal fat and other abdominal tumors [21]. A well-differentiated liposarcoma is characterized by a lesion size of >10 cm, the presence of thick septa, the presence of globular and/or nodular non-adipose areas or masses, and a lesion component of <75% fat [26]. Resection should be considered for huge intra-abdominal lipomatous tumors.

## 4 Conclusion

Liposarcoma of the greater omentum is rare, and 19 cases were reviewed. Differentiation of liposarcoma from other tumors is challenging. Adjuvant therapy has not been established as an effective therapy, and radical resection of the tumor is recommended.

## Funding

This research did not receive any specific grant from funding agencies in the public, commercial, or not-for-profit sectors.

## Ethical approval

This is a case report and it did not require ethical approval from ethics committee. We have got permission from the patient to publish.

## Consent

Written consent to publish this case report was obtained from the patient.

## Author contribution

Shintaro Hashimoto, Junichi Arai, and Hidetoshi Fukuoka were responsible for the study concept and performed the operation. Masato Nishimuta, Hirofumi Matsumoto, Masashi Muraoka, Masahiro Nakashima, and Hiroyuki Yamaguchi collaborated in the patient’s medical care. Hiroyuki Yamaguchi reviewed the manuscript. All authors approved the final article.

## Registration of research studies

Not Applicable.

## Guarantor

Junichi Arai.

## Provenance and peer review

Not commissioned, externally peer-reviewed.

## Declaration of Competing Interest

None.
